# Serum Dipeptidyl Peptidase-4 Activity in Insulin Resistant Patients with Non-Alcoholic Fatty Liver Disease: A Novel Liver Disease Biomarker

**DOI:** 10.1371/journal.pone.0012226

**Published:** 2010-08-18

**Authors:** Gábor Firneisz, Tímea Varga, Gabriella Lengyel, János Fehér, Dóra Ghyczy, Barna Wichmann, László Selmeci, Zsolt Tulassay, Károly Rácz, Anikó Somogyi

**Affiliations:** Department of Medicine, Semmelweis University, Budapest, Hungary; Mayo Clinic College of Medicine, United States of America

## Abstract

**Background:**

In a cross-sectional study we studied the fasting serum DPP-4 enzymatic activity (sDPP-4) and the insulin resistance index (HOMA2-IR) in gliptin naïve patients with type 2 diabetes and in non-alcoholic fatty liver disease (NAFLD) and in healthy controls (CNTRL).

**Methods and Findings:**

sDPP-4 was measured by kinetic assay in 39 NAFLD (F/M:19/20, mean age: 47.42 yrs) and 82 type 2 diabetes (F/M:48/34, 62.8 yrs) patients and 26 (F/M:14/12, 35.3 yrs) controls. Definition of T2D group as patients with type 2 diabetes but without clinically obvious liver disease created non-overlapping study groups. Diagnosis of NAFLD was based on ultrasonography and the exclusion of other etiololgy. Patients in T2D and NAFLD groups were similarly obese. 75 g CH OGTT in 39 NAFLD patients: 24-NGT, 4-IGT or IFG (“prediabetes”), 11-type 2 diabetes. HOMA2-IR: CNTRL: 1.44; T2D-group: 2.62 (p = 0.046 vs CNTRL, parametric tests); NAFLD(NGTonly): 3.23 (p = 0.0013 vs CNTRL); NAFLD(IFG/IGT/type 2 diabetes): 3.82 (p<0.001 vs CNTRL, p = 0.049 vs 2TD group). sDPP-4 activity was higher in NAFLD both with NGT (mean:33.08U/L) and abnormal glucose metabolism (30.38U/L) than in CNTRL (25.89U/L, p<0.001 and p = 0.013) or in T2D groups (23.97U/L, p<0.001 and p = 0.004). Correlations in NAFLD among sDPP-4 and ALT: r = 0.4637,p = 0.0038 and γGT: r = 0.4991,p = 0.0017 and HOMA2-IR: r = 0.5295,p = 0.0026 and among HOMA2-IR and ALT: r = 0.4340,p = 0.0147 and γGT: r = 0.4128,p = 0.0210.

**Conclusions:**

The fasting serum DPP-4 activity was not increased in T2D provided that patients with liver disease were intentionally excluded. The high serum DPP-4 activities in NAFLD were correlated with liver tests but not with the fasting plasma glucose or HbA1C supporting that the excess is of hepatic origin and it might contribute to the speedup of metabolic deterioration. The correlation among γGT, ALT and serum DPP-4 activity and also between serum DPP-4 activity and HOMA2-IR in NAFLD strongly suggests that serum DPP-4 activity should be considered as a novel liver disease biomarker.

## Introduction

Individuals with obesity had 4-fold risk and type 2 diabeticpatients do also have higher risk of dying from cirrhosis than the general population .[1], [2] Vica-versa in the Insulin Resistance Atherosclerosis Study (IRAS) elevated ALT was independently associated with insulin resistance and other findings are also consistent with the role of liver fat in diabetes pathogenesis.[3], [4]

Dipeptidyl peptidase-4 (DPP-4) inactivates both incretin hormones (GIP, GLP-1), therefore DPP-4 inhibitors are used in the treatment of type 2 diabetes.[5] The prevalence of fatty liver disease in a population based study was 34% over 90% of which was due to non-alcoholic causes (NAFLD) that is generally asymptomatic, frequently associated with obesity, type 2 diabetes and metabolic syndrome.[6], [7]

The link between the serum DPP-4 activity and different chronic liver disease, such as the chronic C-virus hepatitis has been previously well established. Higher serum DPP-4 activity was found in patients with chronic C-virus hepatitis, which infectious disease is well known to be associated with diabetes mellitus and insulin resistance.[8], [9] DPP-4 (identical to CD26) is detectable in soluble form in the serum and DPP-4 is widely expressed in the body including bile canaliculi, hepatocytes and hepatic stellate cells (HSCs).[10], [11]

Despite the clear epidemiological associations between the metabolic disorders and chronic liver diseases the exact molecular paths via these clinically important links evolve have not yet been fully elucidated. We first hypothesized that in NAFLD the DPP-4 enzymatic activity is increased which might contribute to the development of type 2 diabetes and metabolic deterioration. The fasting serum DPP-4 enzymatic activity (sDPP-4) was measured in patients with NAFLD and in similarly obese patients with type 2 diabetes but without clinically evident liver disease (defined as T2D group) and also in healthy controls (CNTRL).

## Methods

### Study design and patients

All 147 participants signed the informed consent and this cross-sectional study was approved by the Semmelweis University Regional and Institutional Committee of Science and Research Ethics.

All 39 NAFLD pts were followed by hepatologist and had elevated liver tests for more than 3 months and were diagnosed as having fatty liver by ultrasonography.

All the other possible etiologies such as viral hepatitis, storage diseases including Wilson disease, hemochromatosis and alpha-1 antitrypsin deficiency, autoimmune hepatitis, celiac disease, drug-induced liver disease and rapid loss of weight were excluded. None of these pts had alcohol related disease in the history and none of them had alcohol intake more than 20 g/day. None of the patients had a suggestive family history for a well defined hereditary syndrome and none of the patients presented with seemingly unrelated organ dysfunction.

Although only two out of 39 pts underwent liver biopsy that confirmed the clinical diagnosis and showed macrovesicular steatosis, we applied a non-invasive scoring system to evaluate fibrosis [12] and OGTT (75 gCH) to assess carbohydrate metabolism (except 9 patients with previously known diabetes) in NAFLD. Routine clinical data (including waist circumference), family history, clinical chemistry were recorded.

Separately, 82 type 2 diabetic individuals (23 on insulin therapy) were enrolled from the diabetology outpatient unit, patients with elevated liver tests within 12 months and/or abnormal liver ultrasonography were not eligible for enrolment to this T2D group. Twenty-six volunteers were healthy controls. Homeostasis model assessment (HOMA2-IR) index was calculated using the computer model (http://www.dtu.ox.ac.uk) due to the higher accuracy compared to the HOMA1 method.[13] Metabolic syndrome was both defined as proposed by the NCEP in ATP-III and as recently suggested by the AHA and IDF.[14], [15]

### Clinical characteristics

Clinical data are indicated in table 1.

**Table 1:** Clinical characteristics and the fasting serum DPP-4 activities of healthy individuals (CNTRL) and patients type with 2 diabetes mellitus (2TDM) and non-alcoholic fatty liver disease (NAFLD).

	CNTRL	2TDM	NAFLD *
**No of pts (F/M)**	**26** (12/11)	**82**(48/34)	**39** (19/20)
**Age (y)**	**35.33** (28.76−41.91)	**62.8** (60.4−65.1)	**47.42** (43.83−51)
**BMI (kg/m2)**	**24.49** (22.25−26.73)	**29.77** (28.58−30.96)	**30.36** (29−31.72)
**Abdominal circumference (cm): F/M**	**77.70**(63.4−92)/**96.71** (84.42−109)	**107.6** (102.84−112.34)/**109.91** (104.23−115.58)	**103.73** (95.59−111.88)/**110.56** (105.92−115.20)
**Fasting Plasma Gglucose (mmol/L)**	**4.97** (4.74−5.2)	**8.2** (7.51−8.87)	**6.38** (5.9−6.87)
**HbA1C (%)**	**5.46** (5.33−5.58)	**7.53** (7.18−7.88)	**5.79** (5.54−6.03)
**TG (mmol/L)** ***	**1.12** (0.85−1.38)	**1.94** (1.68−2.2)	**2.41** (1.91−2.91)
**LDL-C (mmol/L)** ****	**2.57** (1.93−3.2)	**2.99** (2.76−3.22)	**3.41** (3.04−3.78)
**ALT (U/L)**	**22.83** (17.40−28.25)	**21.75** (19.46−24.03)	**54.59** (38.49−70.68)
**AST (U/L)**	**26** (21.78−30.24)	**22.64** (20.7−24.59)	**35.13** (28.66−41.59)
**γGT (U/L)**	**23.09** (17.74−28.43)	**31.18** (28.22−34.14)	**100.23** (63.26−137.2)
**HOMA2-IR index**	**1.44** (1.05−1.85)	**2.62** ** (2.22−3.01)	**3.48** (2.97−3.98)
**Fasting Insulin** **	**14.9** (11.8−18)	**17.09** ** (13.79−20.4)	**20.42** (16.58−24.27)
**AST/ALT**	**1.18** (1.0−1.55)	**1.15** (1.05−1.25)	**0.80** (0.70−0.89)
**Metabolic syndrome n/n(%)** *****	0/26 (**0**%) NCEP0/26 (**0**%)	68/82 (**82.9**%) NCEP68/82 (**82.9**%)	21/39 (**54**%) NCEP20/39 (**51**%)
**Fasting serum DPP-4 (U/L)**	**25.89** (24.35−27.43)	**23.97** (22.32−25.61)	**32.15** (29.51−34.80)

Mean values are indicated in bold letters and (95% Confidence Intervals are indicated in parenthesis).

*All NAFLD patients, except 9 previously known diabetic individuals underwent a 75g CH OGTT:

24 NAFLD patients were with normal glucose tolerance (NGT), 4 had either impaired fasting glucose levels or impaired glucose tolerance (“prediabetes”: IFG/IGT), and 11pateint from the NAFLD group were with type 2 diabetes mellitus (28%).

All patients in the 2TDM group were with normal liver tests and normal liver US morphology (patients with such abnormalities were either excluded from the study or re-classified to the NAFLD group).

**Figure 1:**
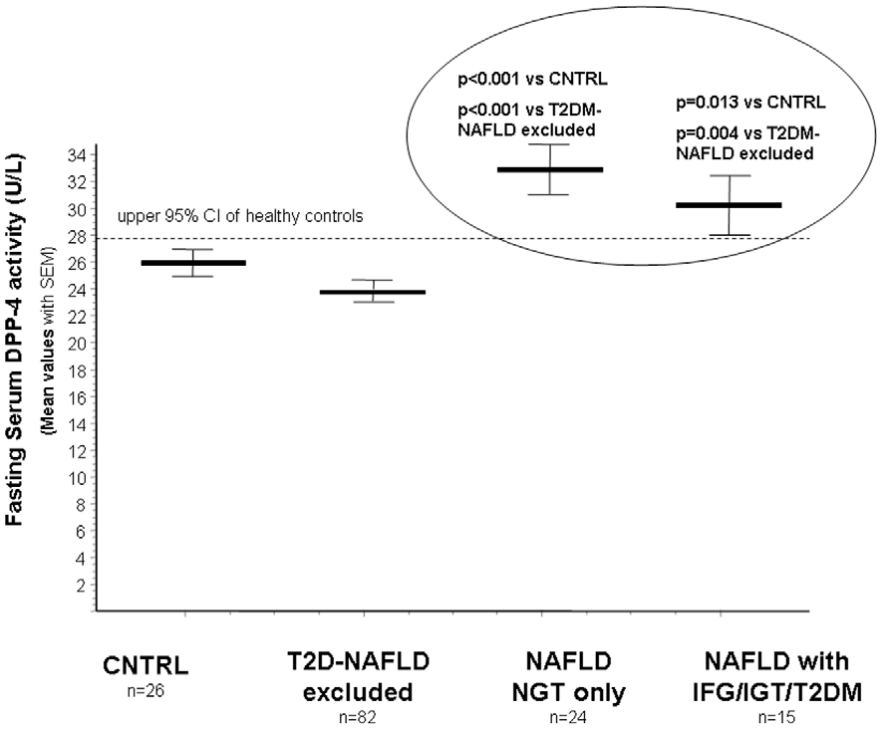
Fasting serum DPP-4 enzymatic activities in patients with type 2 diabetes without clinically diagnosed liver disease (2TD group); in NAFLD patients with normal and abnormal glucose metabolism and in healthy controls (CNTRL group).

In order to generate non-overlapping groups from the point of diabetes we also analyzed separately the DPP-4 activity in patients with NGT and NAFLD that was (33.08 U/L (95%CI:29.66–36.50) significantly higher in comparison with the 2TDM and also compared to the healthy control group. (see figure 1).

In patients with NAFLD a non-invasive fibrosis score was used to assess the degree of intrahepatic fibrosis as suggested and validated by *Angulo et al*. (12) Results are as follows:

No of pts without significant fibrosis (stage 0–2) (NAFLD fibrosis score<−1.455): 29.1.

Indeterminate (NAFLD fibrosis score−1.455–0.676): 6.

No of pts with significant fibrosis (stage 3–4) (NAFLD fibrosis score>0.676): 4.

**23 out of the 82 patients with type 2 diabetes mellitus were treated with insulin – in these cases the HOMA2-IR was calculated using the C-peptide levels.

***Significant differences were found in certain lipid values using ANOVA with the Newman-Kleus post hoc range test in the 39 patients with NAFLD as follows: NAFLD patients also displayed higher triglyceride (TG) levels (2.41mmol/L, 95%CI:2.04–2.78) than in the CNTRL group (1.12mmol/L, 95%CI:0.65–1.58, p<0.001) and the TG levels in the NAFLD group were non-significantly higher than in the T2DM group (1.94, 95%CI: 1.68–2.2).

****The LDL-cholesterol was significantly elevated in NAFLD (3.41mmol/L, 95%CI: 3.05–3.77) compared to controls (2.66mol/L, 95%CI: 2.19–3.13, p = 0.012). The HDL cholesterol was significantly lower in both the T2D group (1.30mmol/L, 95%CI: 1.21–1.39, p<0.05) and the NAFLD group (1.28, 95%CI: 1.15–1.40, p = 0.02) then in the healthy controls (1.54, 95%CI: 1.36–1.69).

*****Metabolic syndrome was both defined as proposed by the NCEP in ATP-III (indicated as NCEP in the table) and as recently suggested by the AHA and IDF the proportion in each study group was calculated using both methods respectively (14, 15).

### Determination of serum DPP-4 activity and other biomarkers

Serum DPP-4 activities were assessed in 39 pts with NAFLD in 82 pts with type 2 diabetes mellitus and in 26 healthy controls. DPP IV activity was determined in a continuous monitoring assay in a microplate reader (Multiskan Plus MK II, Labsystem) at 405 nm, 25°C for 30 min under similar conditions as described earlier.[8], [16] All DPP IV assays were run in duplicates. Fifteen µl serum and 185 µl assay buffer (10 mM Tris-HCl, pH 7.6) containing 2 mmol/L substrate (Gly-Pro-p-nitroanilide tosylate, Gly-Pro-PNA, Bachem, Bubendrof, Switzerland) were pipetted into each microplate wells. Enzyme activity was expressed in nmol/mL/min (U/L) of Gly-Pro-PNA hydrolysed. Aspartate-aminotransferase (AST), alanin-aminotransferase (ALT), gamma-glutamyl-transferase (γGT), alkaline phosphatase (ALP) activities were determined in Olympus AU2700 autoanalyser at 37°C. The enzyme activities are expressed in U/L; bilirubin concentration is given in µmol/L. The index of insulin resistance (IR) was calculated using the HOMA-2 model after fasting insulin and plasma glucose measurements. Specific insulin or C-peptide measurements were made using the Liaison C-peptide and insulin tests on a Liaison analyzer (DiaSorin, Saluggia, Italy).

### Data analysis and statistics

The distribution of data was assessed by the Kolmogorov-Smirnov test. Because we found normal distribution one-way ANOVA with Scheffe's and Newman-Keuls post hoc test and Pearson correlation were used to compare means and asses correlations, p-values lower than 0.05 were evaluated as significant.

## Results

### Clinical data

See in table 1.

### Serum DPP-4 enzyme activity and HOMA2-IR

Fifteen patients out of 39 in the NAFLD group had abnormal glucose metabolism: 11 NAFLD patients presented with type 2 diabetes mellitus and 4 NAFLD patients had (“prediabetes”) either impaired fasting glucose levels or impaired glucose tolerance at the OGTT (75 gCH).

In order to generate non-overlapping groups from the point of diabetes we also analyzed separately the NAFLD patients with normal and abnormal glucose metabolism:

*Fasting serum DPP-4 activity* was higher in NAFLD patients both with NGT (mean:33.08U/L, 95%CI:30.47–35.69) and abnormal glucose metabolism (mean:30.38U/L, 95%CI:27.08–33.68) than in controls (25.89U/L,95%CI: 23.38–28.4, Newman-Kleus p<0.001 and p = 0.013) or in the T2DM (&NAFLD clinically excluded) group (23.97U/L,95%CI: 22.32–25.6 p<0.001 and p = 0.004, respectively). (Figure 1).

**Figure 2:**
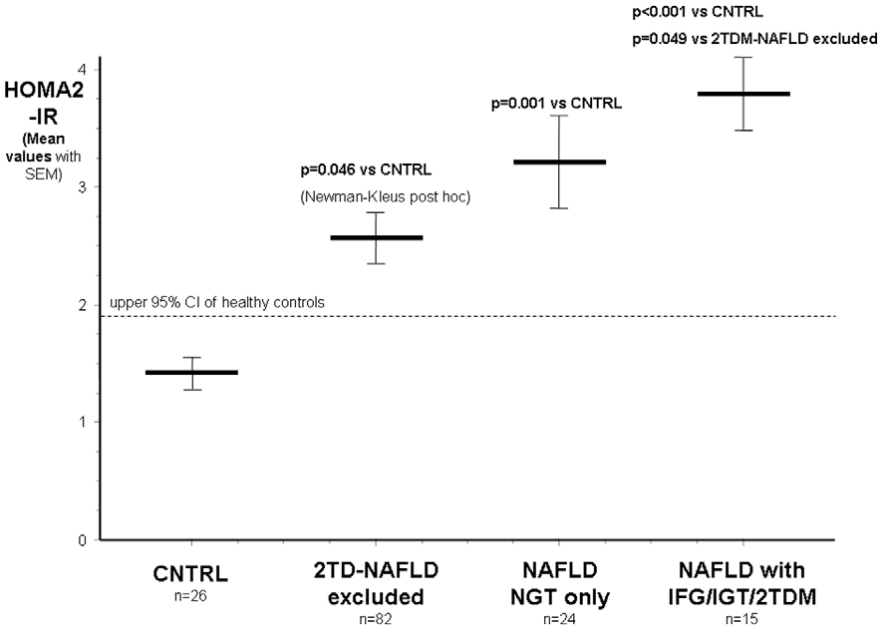
HOMA2-Insulin resistance index in patients with type 2 diabetes without clinically diagnosed liver disease (2TD group), in NAFLD patients with normal and abnormal glucose metabolism and in healthy controls (CNTRL group).

An interesting step-by-step increase was observed in the *HOMA2-IR*:

Controls: mean: 1.44 (95%CI: 1.05–1.85) - T2D-(NAFLD excluded): 2.62 (95%CI: 2.22–3.01, p = 0.046 vs CNTRL) - NAFLD with NGT only: 3.23 (95%CI:2.44–4.01, p = 0.0013 vs CNTRL) and NAFLD with IFG/IGT/2TDM: 3.82 (95%CI:3.19–4.44, p<0.0001 vs CNTRL, p = 0.049 vs 2TD group). (Figure 2).

### Correlation among the serum DPP-4 activity, liver tests and HOMA2-IR in NAFLD

**Figure 3:**
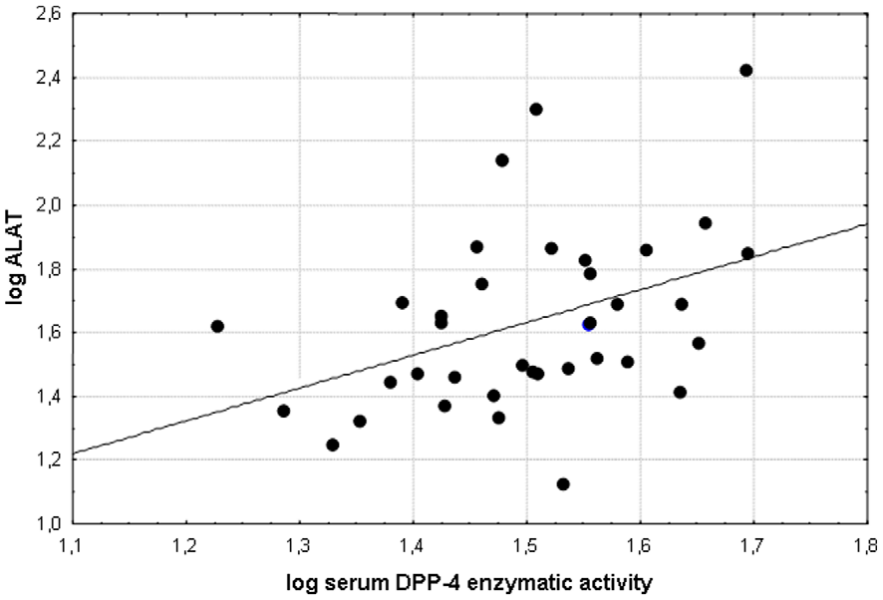
Significant correlation (r = 0.4087; p = 0.0120) between the liver disease biomarker ALT and the fasting serum DPP-4 enzymatic activity after logarithmic transformation in NAFLD patients.

**Figure 4:**
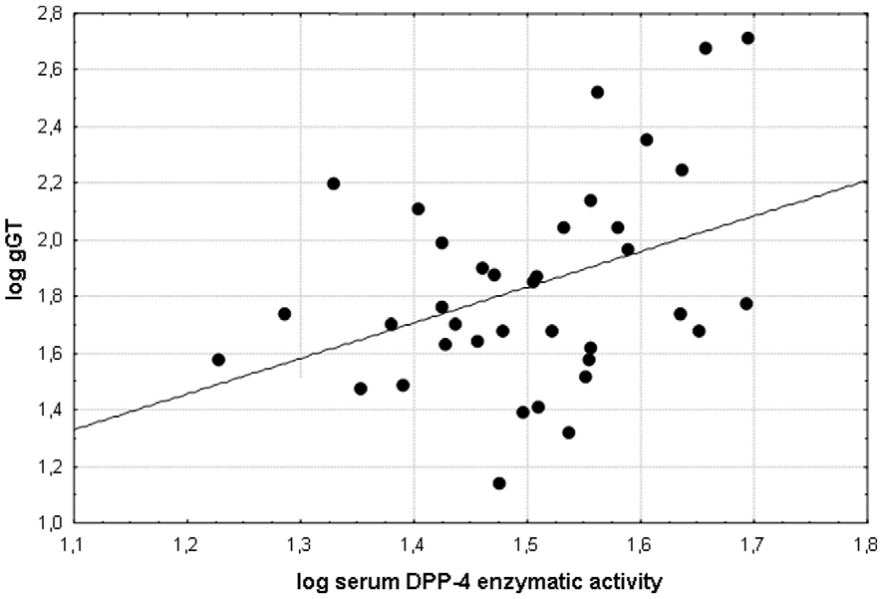
Significant correlation (r = 0.3827; p = 0.019) between the liver disease biomarker γGT and the fasting serum DPP-4 enzymatic activity after logarithmic transformation in NAFLD patients.

**Figure 5:**
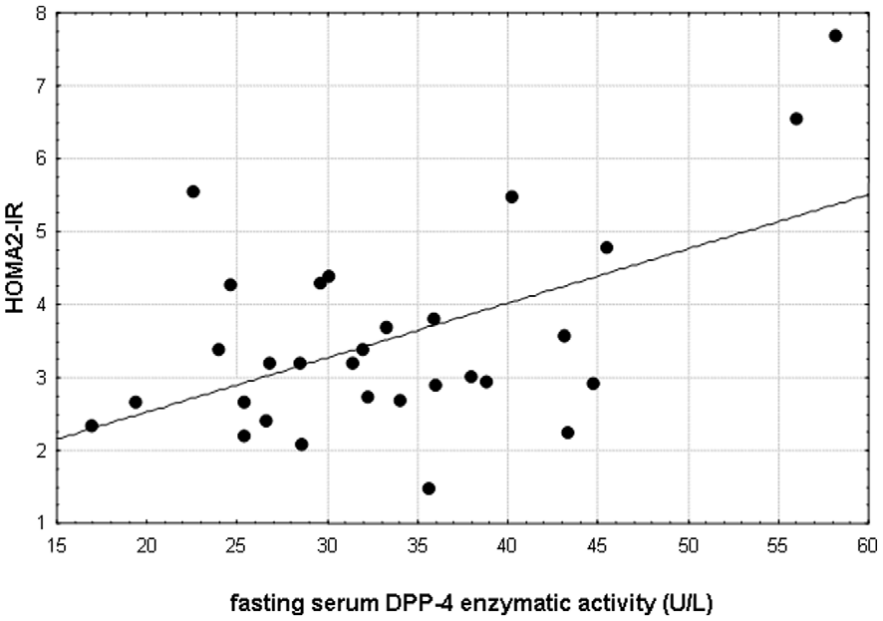
Significant correlation (r = 0.5295; p = 0.0026) between the HOMA2-Insulin resistance index and the fasting serum DPP-4 enzymatic activity in NAFLD patients.

Highly significant correlations were detected among the serum DPP-4 activity and ALT (r = 0.4637 p = 0.0038) and γGT (r = 0.4991, p = 0.0017) values in 39 patients with NAFLD. We performed logarithmic transformation of the liver tests and correlated the values with the logarithmically transformed serum DPP-4 activities (logALT vs logsDPP-4: r = 0.4087; p = 0.0120 and logγGT vs logsDPP-4: r = 0.3827; p = 0.0194-figure 3 and figure 4) these correlations were also significant in the NAFLD group. (Figure 3, Figure 4) Direct correlation was also detected between the serum DPP-4 activity and the HOMA2-IR (r = 0.5295, p = 0.0026) in NAFLD patients. (Figure 5) Further significant correlation was found between the serum DPP-4 activity and the ALP values in NAFLD patients, however this was a magnitude less significant (r = 0.3379, p = 0.0408) than the ones described above.

The HOMA2-IR values were also directly correlated with the ALT (r = 0.4340, p = 0.0147) and γGT (r = 0.4128, p = 0.0210,) but not with the AST or ALP in NAFLD patients. No further correlation was found in this set of NAFLD patients. There was neither correlation between the serum DPP-4 activity and the HbA1C values nor between the DPP-4 activity and the fasting plasma glucose in the T2D group.

### Multiple Logistic Regression

**Figure 6:**
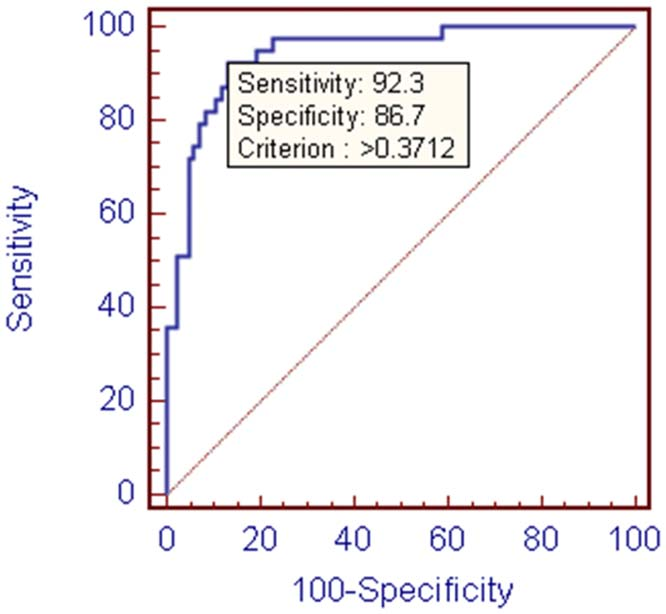
ROCurve for Multiple Logistic Regression (Equation  = 0.7049−0.023*(Fasting Plasma Glucose)+0.0188*(HOMA2-IR)−0.084*(HbA1c)+0.0092*(sDPP−4)+0.004*(ASAT)+0.0000072*(ALAT)+0.0004*(gGT)−0.2859*(ASAT/ALAT)+0.0819*(TG). Criterion 0.3712, below: T2D, above: NAFLD).

Logistic regression was applied to create a formula employing 9 predictive factors (serum DPP-4, fasting plasma glucose, HOMA2-IR, HbA1c, ALAT, ASAT, ASAT/ALAT, gGT, TG) that separates patients in the NAFLD group from those in the T2D group with 92.3% sensitivity and 86.7% specificity as indicated in the ROCurve on Figure 6.

Multiple Logistic Regression Equation  = 0.7049−0.023*(Fasting Plasma Glucose)+0.0188*(HOMA2-IR)−0.084*(HbA1c)+0.0092*(sDPP−4)+0.004*(ASAT)+0.0000072*(ALAT)+0.0004*(gGT)−0.2859*(ASAT/ALAT)+0.0819*(TG). (Criterion 0.3712, below: T2D, above: NAFLD) In addition there was a trend towards higher ALP levels reflected by the number of individuals by ALP quartiles and above increasing form healthy individuals through the T2D to the NAFLD groups.

### Medications applied in the study

Although differences were recorded between the study groups in the medication applied (Metformin, Statins, Glitazons: NAFLD: 12.8%, 35.9%, 2.6%; T2D: 68.3%, 45.1%, 2.4% respectively) these are neither likely to have a major impact on the NAFLD group due to the low rate of biguanid use nor could explain the differences found between the groups due to the similar rate of statin and glitazon use. No difference was found between the metformin taking and non-taking patients in the T2D group in the serum DPP-4 activity (Metformin non-taking: 25.98 U/L (95%CI: 21.39–30.58) Metformin taking: 23.67 U/L (95%CI: 21.82–25.51).

## Discussion

In this cross-sectional study fasting serum dipeptidyl peptidase 4 activity and insulin resistance were assessed in two selected groups of patients, one with non-alcoholic fatty liver disease and the other with type 2 diabetes mellitus without clinically evident liver disease and all results were compared to that of healthy controls (table 1). DPP-4 inhibitor (Gliptin) naïve diabetic patients were subjected to scrutiny due to the success of DPP-4 inhibitors in the treatment of type 2 diabetes mellitus that leads to improved glycemic control by stimulating insulin secretion and biosynthesis, B-cell proliferation and by inhibiting glucagon release and B-cell apoptosis. [5] Despite the large number of clinical trials relatively low number of data were available on the serum DPP-4 enzyme activity prior the initiation of inhibitor therapy. [17], [18] In addition that NAFLD is frequently associated with type 2 diabetes, obesity and the metabolic syndrome higher serum DPP-4 activities were reported in chronic liver diseases with other origin. [8], [9] We presumed that in patients with NAFLD the serum DPP-4 activity would be increased and -via the dysfunctional enetro-insular axis- it might contribute to the impairment of glucose tolerance and the speedup of metabolic deterioration observed in NAFLD. [3]

Surprisingly serum DPP-4 activity was not increased in the T2D group provided that patients with clinically obvious liver disease were intentionally excluded from this study group. In addition we could neither confirm the correlation between sDPP-4 and HbA1C values nor between sDPP-4 and fasting plasma glucose that were reported earlier in type 2 diabetes with smaller sample sizes. [16], [17] Thus, we think that the increment of serum DPP-4 activity reported earlier in patients with type 2 diabetes by others might also be due to the uncounted or unrecognized liver disease. [16], [17]

Conversely the sDPP-4 showed correlation with liver tests (ALT, γGT, and less significantly ALP) in NAFLD (Figure 3, 4), such as in previous reports on chronic liver diseases with different aetiology. [8], [9] Therefore the single publication with lower number of non-alcoholic steatohepatitis patients the serum DPP-4 activity is likely to be influenced by a fundamental bias due to the lack of correlations between the serum DPP-4 activity and liver tests.[18]

The positive correlation found among γGT, ALT and serum DPP-4 activities in NAFLD supports that the excess DPP-4 found in the serum is of hepatic origin (Figure 3, 4). When we analyzed two NAFLD subgroups separately (with normal and abnormal glucose metabolism, respectively) and compared the sDPP-4 to that of the CNTRL and T2D groups (in which liver disease was clinically excluded) we concluded that it is the presence of the (fatty) liver disease that has a primary impact on the serum DPP-4 enzymatic activity and not the hyperglycemia alone. (Figure 1) Vica versa - a significant role of DPP-4 in the hepatic glucose metabolism is further supported by the recent study of Edgerton et al. demonstrating that during vildagliptin and GLP-1 co-treatment the net hepatic glucose uptake was 3-fold greater in the DPP-4 inhibitor treated group than in the control group that was only treated with portal vein GLP-1 infusion but not with the DPP-4 inhibitor and this effect was greater than that predicted by the change in insulin. In this phenomenon the glucagon-lowering effect of DPP-4 vildagliptin must also be taken into consideration [19] In addition this increased serum DPP-4 activity in NAFLD could not be explained by the degree of obesity and (Table 1) the existing correlation between serum DPP-4 activity and insulin resistance (HOMA2-IR) in NAFLD is not surprising, provided that serum DPP-4 activity is considered as a novel liver disease biomarker. (Figure 5)

As published recently by Tabak et al [20] using HOMA method the insulin sensitivity starts to decline earlier (based on his figures-approximately 4 years before the diabetes onset) throughout the course in the development of type2 diabetes mellitus than the definitive impairment of the glucose tolerance. In addition HOMA mostly describes hepatic insulin resistance and steady-state insulin secretion due to the calculation from the fasting plasma glucose and insulin values. Nevertheless the HOMA2-IR calculation might reflect an underestimation of insulin resistance in the T2D group due to the fact that majority of our patients was on metformin therapy and due to the calculation method in this study group. Taken this information together with our observation that NAFLD patients with normal glucose tolerance have similar HOMA2-IR values than those with type 2 diabetes, but without liver disease we would rather interpret this phenomenon as a balanced insulin resistant state, in which the higher insulin secretion and better Beta cell function in NAFLD patients overcomes the already increased insulin resistance compared to the T2D group where a significant B-cell dysfunction could be detected. To prove this explanation we have compared HOMA-B values of NAFLD patients to that of the T2D group and found significantly higher values (NAFLD: 127.3%±49.5 vs T2D: 67.8%±SD40.9 p<0.0001, two tailed T-test). Our cross sectional study might not directly explore the order of events leading to the development of type 2 diabetes mellitus due to the lack of a follow-up period.

Although the membrane bound form of DPP-4 which preponderantly degrades incretin hormones in a local action could not have been assessed in our study, the serum DPP-4 enzymatic activity might be responsible up to 15% of the incretin hormone inactivation process. [5], [21] We must also mention that healthy volunteers were not appropriately age matched controls for type 2 diabetic patients, therefore the influence of ageing on the serum DPP-4 enzyme activity should also be taken into consideration.

The step-by-step increase observed in the HOMA2-IR (Figure 2) with a sequence of.

Healthy controls < T2DM-(if NAFLD excluded) < NAFLD with NGT only < uttermost in NAFLD patients who also presented with abnormal glucose metabolism raises a number of pathophysiological questions. In patients with obesity and 2TDM but without clinically diagnosed liver disease the deterioration of carbohydrate metabolism especially in comparison with the subgroup of NAFLD patients with normal glucose tolerance, but with higher HOMA2-IR values might not only be due to the higher insulin resistance (compared to healthy individuals) but also due to the likely B-cell dysfunction.

Although a statement was published recently for harmonizing the various definitions for the metabolic syndrome [15], in this study 49% of NAFLD patients did not fulfil this criteria of the metabolic syndrome, despite that their HOMA2-IR values were even higher than those with type 2 diabetes but without clinically diagnosed liver disease. Therefore we concluded that even this novel definition might not be evaluated as a perfect harmony since a significant proportion of insulin-resistant patients missed the criteria. In harmony with the data presented here in the IRAS study involving a large cohort, the authors also concluded that the ALT, another liver disease biomarker was independently associated with insulin resistance.[3] These findings are in concordance with the British Women's Heart and Health Study that observed more than a doubling in the risk of incident diabetes in ultrasonography-diagnosed NAFLD and also with the recent French data from the DESIR study that proved that individuals with a BMI of <27 kg/m^2^ gGT is the strongest predictor of diabetes after fasting hyperglycemia. [4], [22]

Based on these results, DPP-4 inhibitors might offer an alternative in the potential prevention of further metabolic deterioration, especially in NAFLD patients. In addition to the potential metabolic benefits, the impact of such therapy on the liver fibrosis might also be in the focus of interest especially because the Fibroblast Activation Protein which is a duplicate molecule of DPP-4 (FAP-DPP-4 shows 88% homology at cDNA level) is present at the tissue remodeling interface on hepatic stellate cells (HSCs, ITO cells) which are thought to primarily produce the accumulating extracellular matrix (ECM) proteins (including collagens) in chronic liver diseases eventually leading to the fibrosis and cirrhosis of the liver.[23]

### Ethics approval

This Study was approved by the Semmelweis University Regional and Institutional Committee of Science and Research Ethics.
